# Cystic nodal metastasis in patients with oropharyngeal squamous cell carcinoma receiving chemoradiotherapy: Relationship with human papillomavirus status and failure patterns

**DOI:** 10.1371/journal.pone.0180779

**Published:** 2017-07-07

**Authors:** Yu-Han Huang, Chih-Hua Yeh, Nai-Ming Cheng, Chien-Yu Lin, Hung-Ming Wang, Sheung-Fat Ko, Cheng-Hong Toh, Tzu-Chen Yen, Chun-Ta Liao, Shu-Hang Ng

**Affiliations:** 1 Department of Radiology, Taipei Medical University, Shuang-Ho Hospital, Taipei, Taiwan; 2 Molecular Imaging Center, Chang Gung Memorial Hospital, Linkou, Taiwan; 3 Department of Diagnostic Radiology, Chang Gung Memorial Hospital, Chang Gung University, Kueishan, Taoyuan, Taiwan; 4 Department of Nuclear Medicine, Chang Gung Memorial Hospital, Chang Gung University, Kueishan, Taoyuan, Taiwan; 5 Department of Radiation Oncology, Chang Gung Memorial Hospital, Chang Gung University, Kueishan, Taoyuan, Taiwan; 6 Department of Medical Oncology, Chang Gung Memorial Hospital, Chang Gung University, Kueishan, Taoyuan, Taiwan; 7 Department of Otorhinolaryngology, Head and Neck Surgery, Chang Gung Memorial Hospital, Chang Gung University, Kueishan, Taoyuan, Taiwan; Penn State University School of Medicine, UNITED STATES

## Abstract

**Objectives:**

We investigated the relationships of cystic nodal metastasis, human papillomavirus (HPV) status, and treatment failure patterns in patients with oropharyngeal squamous cell carcinoma (OPSCC) treated with chemoradiotherapy.

**Methods:**

We retrospectively reviewed pretreatment MRI and clinical courses of patients with OPSCC whose tumors were tested for HPV-induced p16 expression via immunohistochemistry and who completed chemoradiotherapy. Cervical cystic nodal metastasis and necrotic nodal metastasis were classified on MRI.

**Results:**

Of 98 patients eligible for analysis, 33 were p16-positive. Cystic nodal metastasis was significantly more prevalent in p16-positive than in p16-negative patients (39.4% versus 18.5%, respectively; *p* = 0.025). Necrotic nodal metastasis was significantly more prevalent in p16-negative than in p16-positive patients (73.8% versus 51.5%, respectively; *p* = 0.027). On multivariate analysis, necrotic nodal metastasis (odds ratio [OR] = 7.310, p = 0.011) was an independent predictor of regional failure, while advanced nodal stage (OR = 4.119, *p* = 0.022) and cystic nodal metastases (OR = 0.087, *p* = 0.026) were independent positive and negative predictors of distant failure, respectively.

**Conclusions:**

Cervical cystic and necrotic nodal metastases are associated with HPV-induced p16-positive and p16-negative OPSCC, respectively. Patients with necrotic nodal metastasis at presentation have an increased risk of regional failure. Distant failure is directly and inversely correlated with advanced nodal stage and cystic nodal metastasis, respectively.

## Introduction

Oropharyngeal squamous cell carcinoma (OPSCC) is one of the most common types of head and neck cancer. Most patients with OPSCC have regional nodal metastasis at presentation and are generally treated with an organ-preservation approach based on chemoradiotherapy [1]. The major risk factors for OPSCC include alcohol consumption, tobacco smoking, and human papillomavirus (HPV) infection [2,3]. HPV-related OPSCC shows a gene expression profile that is distinct from OPSCC related to tobacco or alcohol [4]. HPV-positive patients usually have more favorable prognoses than HPV-negative patients, with superior responses to radiation and chemotherapy [5]. HPV16/18 are the most commonly detected transcriptionally active HPV types. Immunohistochemistry for p16 overexpression has emerged as a robust surrogate biomarker for HPV-mediated carcinogenesis and as an independent positive prognosticator [6]. Direct detection of HPV is not used as a defining factor owing to limited availability of the test, cost, and lack of advantage of over p16 overexpression in terms of predicting survival. Hence, p16 overexpression was chosen as the best identifier of disease because of its low cost, universal applicability, and ease of analysis compared to other HPV identifiers [7]. The association between HPV status and cervical cystic nodal metastasis has been investigated in retrospective reviews of pretreatment computed tomography (CT) examinations of OPSCC [8–10]. Goldenberg et al. [8] classified cystic metastatic nodes and necrotic metastatic nodes on images as follows: cystic nodes were defined as those having homogeneous fluid content without internal complex, irregular, or solid areas and an enhancing capsule <2 mm in thickness, while necrotic metastatic nodes were defined as those having thicker walls and irregular, complex central low attenuation. They found that 87% of the patients (11 of 13) with cystic nodal metastases were HPV-positive; thus, they proposed that a strong association exists between cystic cervical nodal metastases and HPV-positive OPSCC. Subsequently, Cantrell et al. [9] and Morani et al. [10] used these criteria in their review of CT images of their patients with OPSCC; their results supported the existence of an association between cystic nodal metastasis and HPV-positive OPSCC. However, to our knowledge, a correlation between cystic nodal metastasis and tumor control after chemoradiotherapy in patients with OPSCC has not been reported.

Since both cystic and necrotic metastatic nodes appear as nodal low-density lesions on CT, they may be grouped together when interpreting CT results in clinical practice or when conducting research. Indeed, conflicting results have been reported regarding the correlation between intranodal low-density lesions in contrast-enhanced CT and treatment response to chemotherapy in head and neck cancers. Munck et al. [11] and Janot et al. [12] reported that isodense metastatic nodes had a significantly better response to chemotherapy compared to hypodense metastatic nodes; however, Wong et al. [13] indicated that lymph node density was poorly correlated with chemotherapy, and attributed the discordant results to the fact that CT cannot truly reflect viable tumor burden in lucent areas of metastatic lymph nodes. As MRI has better contrast resolution than CT, it may better differentiate between cystic and necrotic metastatic nodes by revealing any subtle internal complex, irregular, or solid foci in some seemingly homogeneous intranodal low-attenuation nodes on CT. In this study, we investigated the relationships of cystic nodal metastasis identified on pretreatment MRI with HPV-induced p16 overexpression, and treatment failure patterns in patients with OPSCC who were treated with chemoradiotherapy.

## Materials and methods

### Patients

We retrospectively reviewed patients with OPSCC diagnosed between 2008 and 2013 at Chang Gung Memorial Hospital, Linkou, Taiwan. The Institutional Review Board of our hospital approved this retrospective cohort study; the requirement for informed patient consent was waived owing to the respective nature of the study. The inclusion criteria were as follows: (1) patients had newly diagnosed, previously untreated OPSCC with regional nodal metastasis; (2) patient samples had been tested for p16 with immunohistochemistry; and (3) patients had completed concomitant chemoradiotherapy with curative intent. The exclusion criteria included patients with distant metastatic disease at presentation and those with a previous history of malignancy. Our patients underwent a work-up before chemoradiotherapy that included their medical history and a complete physical examination, flexible fiber-optic pharyngoscopy, routine blood biochemistry, MRI of the head and neck, chest radiographs, liver ultrasonography, and ^18^F-fluorodeoxyglucose-positron emission tomography (^18^F-FDG PET)/CT.

### MRI

MRI of the head and neck region was performed using a 3 Tesla MRI scanner (Magnetom Trio with TIM, Siemens, Erlangen, Germany). T2-weighted turbo spin echo (TSE) images with fat saturation and T1-weighted TSE images were obtained in the both the axial and coronal projections. Section thickness was 5 mm without interslice gap in the axial projection and 4 mm without interslice gap in the coronal projection. After gadolinium dimeglumine injection at a dose of 0.1 mmol/kg of body weight, we obtained fat-suppressed T1-weighted axial, sagittal, and coronal images sequentially.

Pretreatment MRI scans of our patients were reviewed independently by two neuroradiologists, with 21 and 6 years of experience in head and neck imaging, respectively, who were blinded to the patients' HPV statuses and treatment outcomes. Neck nodes were considered metastatic if intranodal necrosis or cysts were present irrespective of their sizes, if their shortest axial diameter reached 10 mm with strong uptake on ^18^F-FDG PET/CT, or if cytology findings on ultrasound-guided fine needle aspiration were positive. On MRI, a cystic nodal metastasis was defined as a metastatic node with a thin (<2 mm) enhancing capsule and central homogeneous fluid-like signal intensity, or with a focal intra-nodal homogeneous cystic focus in which over 70% of the margin circumference was well defined and smooth. A necrotic metastatic node was defined as having a thick or irregular wall comprising >30% of the circumference, or having complex fluid-like content with heterogeneous intensities.

### p16 immunohistochemistry

p16 immunohistochemistry was performed on samples biopsied from the primary tumor sites using the CINtec histology kit (MTM laboratories, Heidelberg, Germany) according to the manufacturer’s protocol. High p16 expression was defined when nuclear and cytoplasmic staining was strong (intensity >3) plus the distribution was diffuse (≥75% of the tumor specimen).

### Treatment and follow-up

All participants received intensity-modulated radiotherapy using 6 MV photon beams generated by linear accelerators. Shrinkage field technique with 2 Gy per single daily fraction, 5 days a week, was performed. The initial prophylactic field included the gross tumor with at least 1 cm margins and neck lymphatics at risk for 46–56 Gy, following which cone down boost to the initial gross tumor area with reduced margins to 72 Gy. Bilateral neck prophylaxis was usually performed for patients with locally advanced disease. Ipislateral neck irradiation field encompassed whole neck lymphatics in all patients with cN+ status. Contralateral neck prophylactic field usually encompassed level II–III for tonsil cancers, level Ib–III for soft palate cancers, and level II–IV for tongue base cancers depending on tumor origin and extension. Concomitant chemotherapy consisted of intravenous cisplatin 50 mg/m^2^ on day 1, and oral tegafur 800 mg/day plus oral leucovorin 60 mg/day from day 1 to day 14. This regimen was repeated every 2 weeks throughout the radiotherapy course [14]. Following treatment, all patients underwent a clinical follow-up examination every 1–3 months. A baseline posttreatment MRI was performed at 3 months after completion of chemoradiotherapy. Follow-up MRI or CT was performed every 6 months thereafter or upon clinical deterioration. After completion of primary chemoradiotherapy, patients with a primary tumor present at the site of origin were classified as having local failure, those with metastatic neck lymph nodes were classified as having regional failure, and those with metastatic disease beyond regional lymph nodes were classified as having distant failure. Whenever possible, surgical- or image-guided biopsies were performed in all patients in whom failure was suspected. Patients without pathologically proven failure were followed for at least 24 months after treatment or until death.

### Statistical analysis

The clinical characteristics of the enrolled patients, as well as imaging characteristics of metastatic nodal diseases, were compared in patients with and without p16 overexpression using the Fisher exact test and Pearson’s chi-square test. Cohen’s kappa coefficient was used to assess the inter-rater agreement for cystic and necrotic nodal metastases between two observers. Univariate analysis comparing clinical and imaging characteristics to treatment failure patterns were also performed using the Fisher exact test and Pearson’s chi-square test. All factors found to be significant on univariate analyses were analyzed using a multivariate logistic regression model to identify independent predictors. A two-tailed *p*-value less than 0.05 was deemed to be significant in all statistical analyses. The SPSS Statistics 20 software (SPSS Inc., Chicago, IL, USA) was used for all analyses.

## Results

A total of 105 patients who were newly diagnosed with OPSCC and who met the inclusion criteria were enrolled in the study. Seven patients died before definitive treatment failure could be determined; therefore, 98 patients were available for analysis, including 89 men and 9 women with a median age of 53 years (range 33–78 years). The palatine tonsil was the most common primary tumor site, occurring in 62 patients (63.3%). All our patients had advanced stage disease based on 8th edition of American Joint Committee on Cancer staging (2017); 10 patients (10.2%) had stage I, 6 (6.1%) had stage II, 22 (22.4%) had stage III, 37 (37.8%) had stage IVa, and the remaining 23 (23.5%) had stage IVb. The clinicopathological characteristics of the 98 patients are summarized in Table 1. Of these 98 patients, 33 were p16-positive, including 28 men and 5 women with a mean age of 55.5 years. The remaining 65 patients were p16-negative, including 61 men and 4 women with a median age of 51.7 years. Comparisons of clinical and imaging characteristics relating to p16 status are presented in Table 2. No significant differences between p16-negative and p16-positive patients were found in terms of sex, age, T-stage, or N-stage. Alcohol, cigarette, and betel quid use were more prevalent in p16-negative patients than in their p16-positive counterparts. The inter-observer agreement between the two raters for both cystic nodal metastases and necrotic nodal metastasis was substantial (Cohen’s kappa for cystic nodal metastasis: k = 0.663, *p*<0.001; and for necrotic nodal metastasis: k = 0.780, *p*<0.001). Cystic nodal metastasis was significantly more prevalent in patients with p16-positive tumors than those with p16-negative tumors (39.4% versus 18.5%, *p* = 0.025). In contrast, necrotic nodal metastasis was significantly more prevalent in patients with p16-negative tumors than those with p16-*p*ositive tumors (73.8% versus 51.5%, *p* = 0.027).

**Table 1:** Clinicopathological characteristics of patients with oropharyngeal squamous cell carcinoma (N = 98).

Characteristics	n (%)
*Sex*	
Female	9 (9.2%)
Male	89 (90.8%)
Age* *(years)*	
≤45	16 (16.3%)
>45	82 (83.7%)
*p16 overexpression*	
No	65 (66.3%)
Yes	33 (33.7%)
*Serum hemoglobin*^$^ *(g/dL)*	
≤12.0	20 (20.4%)
>12.0	78 (79.6%)
*Alcohol consuming*	
No	28 (28.6%)
Yes	70 (71.4%)
*Cigarette smoking*	
No	24 (24.5%)
Yes	74 (75.5%)
*Betel quid chewing*	
No	43 (43.9%)
Yes	55 (56.1%)
*Subsite*	
Palatine tonsil	62 (63.3%)
Tongue base	21 (21.4%)
Soft palate	4 (4.1%)
Multiple subsites	11 (11.2%)
*T-stage*	
1	3 (3.0%)
2	24 (24.5%)
3	13 (13.3%)
4	58 (59.2%)
*N-stage*	
1	29 (29.6%)
2	53 (54.1%)
3	16 (16.3%)
*AJCC stage*	
I	10 (10.2)
II	6 (6.1)
III	22 (22.4%)
IVa	37 (37.8%)
IVb	23 (23.5%)
*Differentiation*	
Well	5 (5.1%)
Moderately	48 (49.0%)
Poorly	36 (36.7%)
Unavailable	9 (9.2%)

* Age (years): range: 33–78; mean: 52.98; median: 53.

^$^ Serum hemoglobin level: range, 6.1–17.4; mean: 13.71; median: 13.90.

AJCC: American Joint Committee on Cancer.

**Table 2:** Clinical and imaging parameters of patients with oropharyngeal squamous cell carcinoma according to p16 status (N = 98).

Parameters	p16-positive (n = 33) N (%)	p16-negative (n = 65) N (%)	*p*-*value*
*Sex*			0.159 ^&^
Female (N = 9)	5 (15.2%)	4 (6.2%)	
Male (N = 89)	28 (84.8%)	61 (93.8%)	
*Age (years)*			0.050
≤45 (N = 16)	2 (6.1%)	14 (21.5%)	
>45 (N = 82)	31 (93.9%)	51 (78.5%	
*Alcohol consuming*			0.002*
No (N = 28)	16 (48.5%)	12 (18.5%)	
Yes (N = 70)	17 (51.5%)	53 (81.5%)	
*Cigarette smoking*			0.001*
No (N = 24)	15 (45.5%)	9 (13.8%)	
Yes (N = 74)	18 (54.5%)	56 (86.2%)	
*Betel quid chewing*			<0.001*
No (N = 43)	23 (69.7%)	20 (30.8%)	
Yes (N = 55)	10 (30.3%)	45 (69.2%)	
*Serum hemoglobin level*			0.286
≤13.7 (N = 46)	13 (39.4%)	33 (50.8%)	
>13.7 (N = 52)	20 (60.6%)	32 (49.2%)	
*Advanced T-stage*^#^			0.062
No (N = 27)	13 (39.4%)	14 (21.5%)	
Yes (N = 71)	20 (60.6%)	51 (78.5%)	
*Advanced N-stage*^$^			0.102
No (N = 51)	21 (63.6%)	30 (46.2%)	
Yes (N = 47)	12 (36.4%)	35 (53.8%)	
*Necrotic nodal metastasis*			0.027*
No (N = 33)	16 (48.5%)	17 (26.2%)	
Yes (N = 65)	17 (51.5%)	48 (73.8%)	
*Cystic nodal metastasis*			0.025*
No (N = 73)	20 (60.6%)	53 (81.5%)	
Yes (N = 25)	13 (39.4%)	12 (18.5%)	

* *p*-value <0.05, indicating statistical significance.

^&^ Calculated by Fisher’s exact test.

^#^ Advanced T-stage: T3–4 in HPV positive patients and T3–T4b in HPV negative patients.

^$^ Advanced N-stage: Contralateral or bilateral nodal metastases or nodal metastasis >6 cm in all patients, or extranodal extension in p16-negative patients.

**Fig 1:**
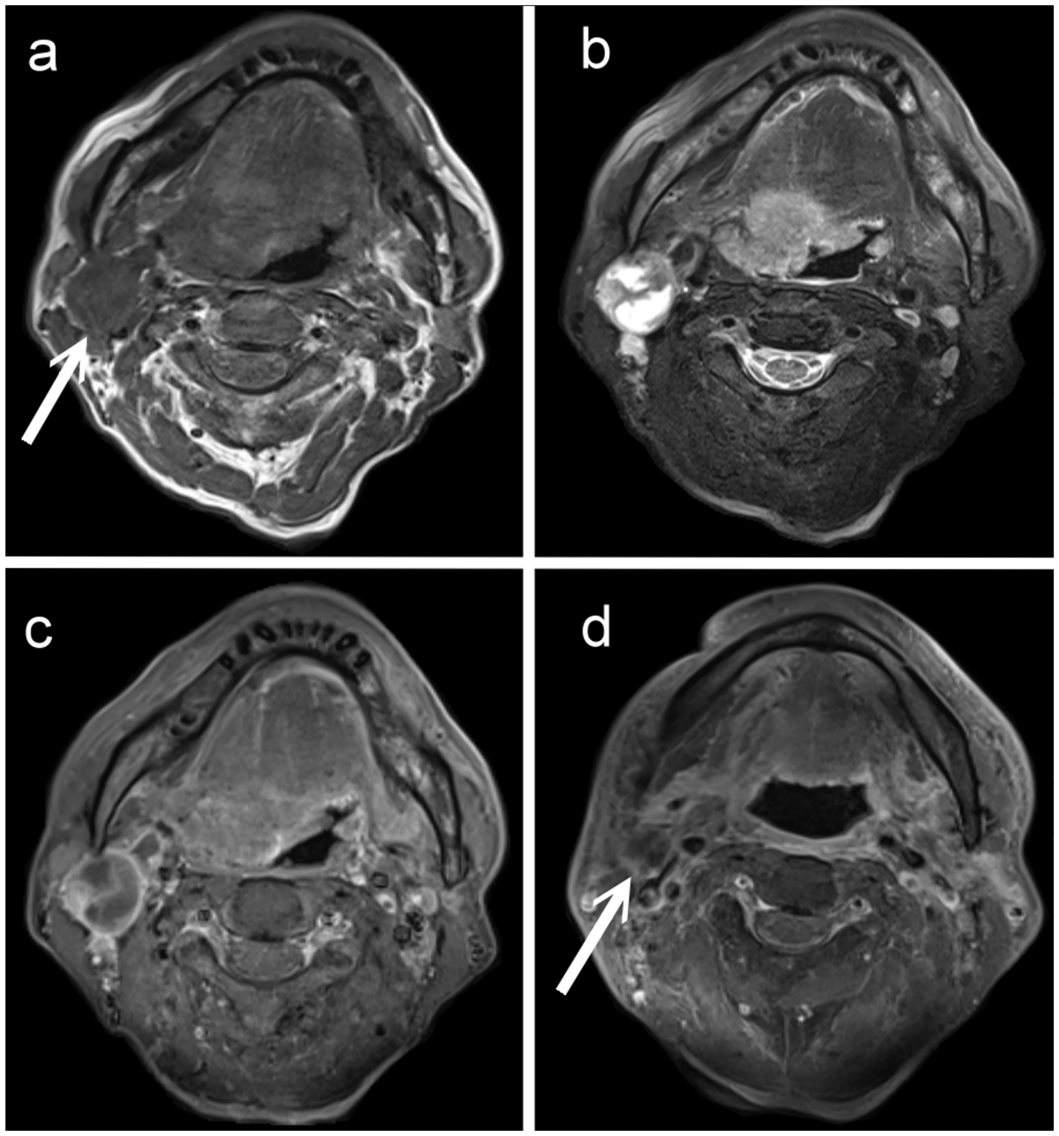
A 54-year-old male patient with right tongue base oropharyngeal squamous cell carcinoma and necrotic nodal metastasis. (A) T1-weighted, (B) fat-suppressed T2-weighted, and (C) contrast-enhanced fat-suppressed T1-weighted MRI images show an enlarged necrotic lymph node (arrow) in the right neck level IIA with irregular walls and central complex fluid-like content. (D) Contrast-enhanced fat-suppressed T1-weighted MRI three months after completion of chemoradiotherapy shows a residual necrotic node (arrows). Subsequent echo-guided fine needle aspiration cytology yielded metastatic carcinoma. The patient also experienced lung metastasis at a later date.

**Fig 2:**
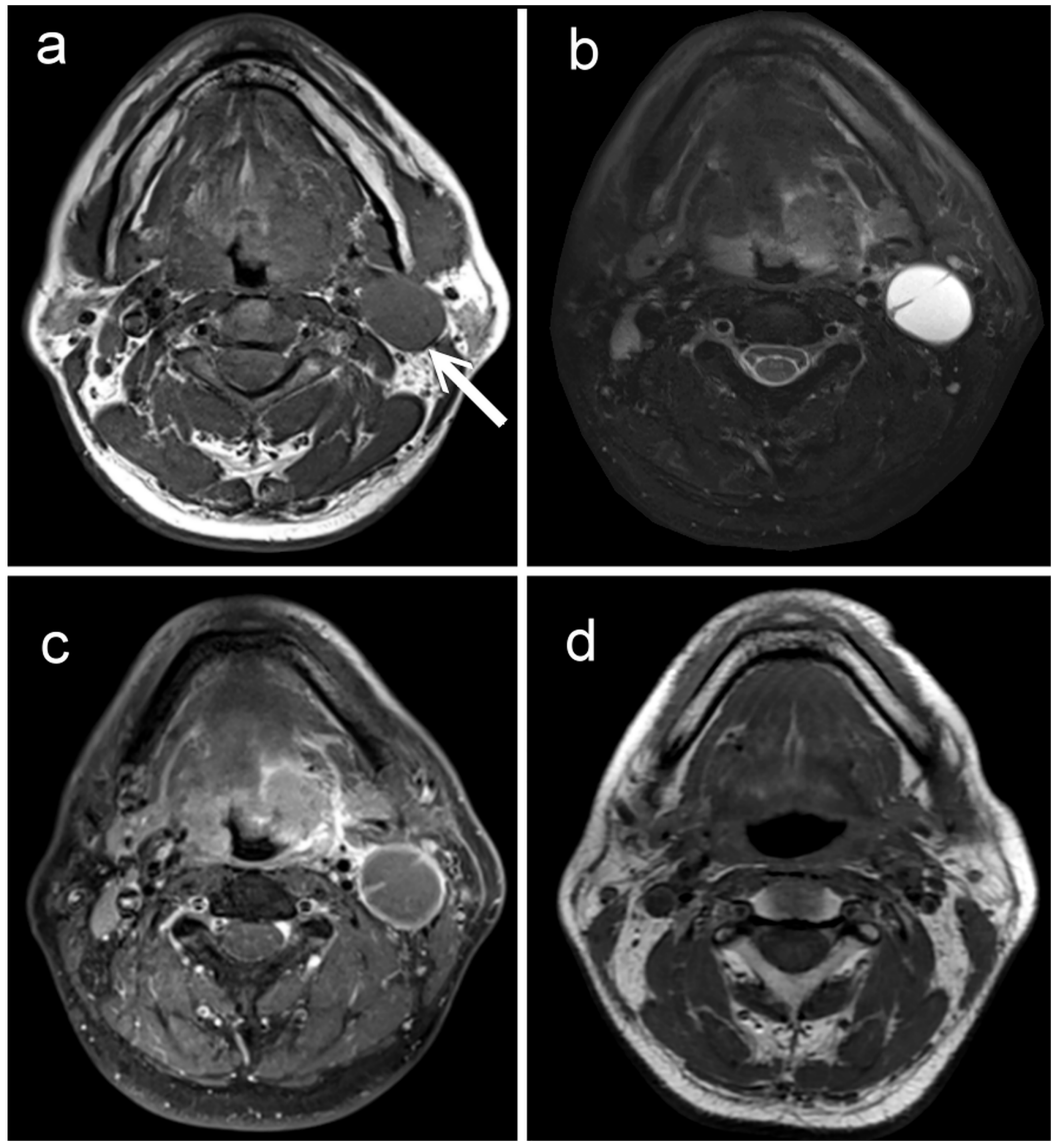
A 46-year-old male patient with left tongue base oropharyngeal squamous cell carcinoma and cystic nodal metastasis. (A) T1-weighted, (B) fat-suppressed T2-weighted, and (C) contrast-enhanced fat-suppressed T1-weighted MRI images show an enlarged cystic lymph node (arrow) in the left neck level IIA with a thin, well-defined smooth margin, and central homogeneous fluid content. (D) T1-weighted MRI three months after completion of chemoradiotherapy shows regression of the neck node. The patient was disease-free during three years of clinical and imaging follow-up.

After a median follow-up time of 34 months (range 6–65 months), 38 of the 98 patients (38.8%) had treatment failure, including 4 (4.0%) with local failure, 7 (7.1%) with regional nodal failure, 9 (9.2%) with distant failure, 7 (7.1%) with both local and regional failure, 2 (2.0%) with both regional and distant failure, and 9 (9.2%) with local, regional, and distant failure. The most common site of distant failure was the lung (16 patients), followed by the bone (7 patients), liver (2 patients), and mediastinal nodes (1 patient). On univariate analysis (Table 3), advanced pretreatment T-stage (T3–4 in p16-positive patients or T3–T4b in p16-negative patients) was the only significant factor influencing local control; neither necrotic nor cystic nodal metastases were correlated with local failure at the primary site. The relative risk of regional failure was significantly higher in patients with necrotic nodal metastases than in those without (risk ratio [RR] = 5.839, *p* = 0.002) (Fig 1), but was significantly lower in patients with p16-positive status than in those with p16-negative status (RR = 0.375, *p* = 0.030). The relative risk of regional failure was also lower in patients with cystic nodal metastases (Fig 2), but the difference was not significant. The relative risk of distant failure was significantly higher with advanced pre-treatment N-stage (contralateral or bilateral nodal metastases or nodal metastasis >6 cm in all patients, or extranodal extension in p16-negative patients; RR = 3.255, p = 0.007) and necrotic nodal metastasis (RR = 4.541, *p* = 0.012), but was significantly lower with cystic nodal metastasis (RR = 0.15, *p* = 0.018) and p16-positive status (RR = 0.347, *p* = 0.048). A multivariate logistic regression model (Table 4) showed that necrotic nodal metastasis (odds ratio [OR] = 7.310, *p* = 0.011) was an independent predictor of regional failure, while advanced N-stage (OR = 4.119, *p* = 0.022) and cystic nodal metastases (OR = 0.087, *p* = 0.026) were positive and negative independent predictors of distant failure, respectively.

**Table 3:** Univariate analysis of failure patterns in patients with oropharyngeal squamous cell carcinoma (N = 98).

Parameters	Local failure			Regional failure			Distant failure		
	Events n (%)	Risk ratio	*p*-value	Events n (%)	Risk ratio	*p*-value	Events n (%)	Risk ratio	*p*-value
*Sex*		N/A	0.111^&^		2.427	0.441^&^		1.919	0.681^&^
Female (N = 9)	0 (0.0%)			1 (11.1%)			1 (11.1%)		
Male (N = 89)	20 (22.5%)			24 (27.0%)			19 (21.3%)		
*Age*		0.780	0.735^&^		1.431	0.755^&^		1.760	0.513^&^
≤45 (N = 16)	4 (25.0%)			3 (18.8%)			2 (12.5%)		
>45 (N = 82)	16 (19.5%)			22 (26.8%)			18 (22.0)		
*p16*		0.492	0.147		0.375	0.030*		0.347	0.048*
Negative (N = 65)	16 (24.6%)			21 (32.3%)			17 (26.2%)		
Positive (N = 33)	4 (12.1%)			4 (12.1%)			3 (9.1%)		
*Hemoglobin*		0.724	0.418		0.600	0.129		0.724	0.418
≤13.7 (N = 46)	11 (23.9%)			15 (32.6%)			11 (23.9%)		
>13.7 (N = 52)	9 (17.3%)			10 (19.2%)			9 (17.3%)		
*Alcohol*		2.271	0.132		2.100	0.107		3.620	0.052^&^
No (N = 28)	3 (10.7%)			4 (14.3%)			2 (7.1%)		
Yes (N = 70)	17 (24.3%)			21 (30.0%)			18 (25.7%)		
*Smoking*		2.928	0.144^&^		1.703	0.253		1.840	0.385^&^
No (N = 24)	2 (8.3%)			4 (16.7%)			3 (12.5%)		
Yes (N = 74)	18 (24.3%)			21 (28.4%)			17 (23.0%)		
*Betel quid*		1.172	0.695		1.661	0.166		1.821	0.161
No (N = 43)	8 (18.6%)			8 (18.6%)			6 (14.0%)		
Yes (N = 55)	12 (21.8%)			17 (30.9%)			14 (25.5%)		
Advanced *T-stage*^#^		N/A	0.002*		2.000	0.134		2.153	0.159
No (N = 27)	0 (0.0%)			4 (14.8%)			3 (11.1%)		
Yes (N = 71)	20 (28.2%)			21 (29.6%)			17 (23.9%)		
*Advanced N-stage*^$^		2.015	0.087		1.929	0.063		3.255	0.007*
No (N = 52)	7 (13.7%)			9 (17.6%)			5 (9.8%)		
Yes (N = 46)	13 (27.7%)			16 (34.0%)			15 (31.9%)		
*Necrotic node*		1.520	0.358		5.839	0.002*		4.541	0.012*
No (N = 38)	5 (15.2%)			2 (6.1%)			2 (6.1%)		
Yes (N = 78)	15 (23.1%)			23 (35.4%)			18 (27.7%)		
*Cystic node*		0324	0.074		0.398	0.073		0.154	0.018*
No (N = 87)	18 (24.7%)			22 (30.1%)			19 (26.0%)		
Yes (N = 29)	2 (8.0%)			3 (12.0%)			1 (4.0%)		

* p value <0.05, indicating statistical significance.

^&^ Calculated by Fisher’s exact test.

^#^ Advanced T-stage: T3–4 in p16-positive patients and T3–T4b in p16-negative patients.

^$^ Advanced N-stage: Contralateral or bilateral nodal metastases or nodal metastasis >6 cm in all patients, or extranodal extension in p16-negative patients.

**Table 4:** Multivariate analysis of failure patterns in patients with oropharyngeal squamous cell carcinoma.

Parameters	Regional nodal recurrence		Distant metastases	
	Odds ratio	*p*-value	Odds ratio	*p*-value
*p16 overexpression*	0.372	0.111	0.635	0.545
*Advanced N-stage*	-	-	4.119	0.022*
*Necrotic nodal metastasis*	7.310	0.011*	4.579	0.065
*Cystic nodal metastasis*	-	-	0.087	0.026*

* *p*-value <0.05 indicating statistical significance.

## Discussion

Although head and neck squamous cell carcinoma (HNSCC) incidences have remained stable or even declined in recent years, OPSCC rates have increased because of higher HPV infection rates [3]. HPV-positive tumors represent a separate subset of OPSCC with a unique epidemiology; their etiologies and biologic characteristics are distinct, and they generally exhibit more favorable prognoses [2,3,5,15]. Patients with HPV-related OPSCC tend to be relatively young and are less exposed to tobacco and alcohol; these are distinguishing factors compared to patients with classic HPV-unrelated OPSCC [15,16]. In our Taiwanese study population, we found that, in addition to alcohol-drinking and tobacco-smoking, betel quid chewing was found to occur significantly more frequently among HPV-induced p16-negative patients with OPSCC compared to their p16-positive counterparts. Indeed, betel quid is claimed to be the most important contributing factor to the increasing incidence rates of OPSCC in Taiwan besides alcohol and tobacco [17]. Our results indicate that increased rates of OPSCC due to betel quid use predominantly represent p16-negative disease. Another noteworthy clinical characteristic found in this study was the relatively older age of our p16-positive patients, with a mean of 56 years. We posit that this may be attributable, at least in part, to differences in ethnicity, lifestyle, and/or sexual behavior between Taiwanese and Western populations.

A significant association between HPV infection and cystic cervical nodal metastasis in patients with OPSCC was initially proposed by Goldenberg et al., [8] who reported that the majority of patients with OPSCC and cystic nodal metastases were HPV-positive. Thereafter, Cantrell et al. [9] and Morani et al. [10] affirmed this association by showing that cystic nodal metastasis was significantly more prevalent in HPV-positive patients than HPV-negative patients after reviewing CT images of their OPSCC patients (36% versus 9%, and 38.4% versus 26%, respectively). Morani et al. even claimed that intranodal cystic foci observed on pretreatment CT appear to be a potential radiological signature strongly associated with the HPV status of patients with OPSCC. In this study, we distinguished cystic metastatic nodes from necrotic metastatic nodes by using 3 Tesla MRI, which has a higher tissue contrast resolution than CT. Our results still showed a significantly higher prevalence rate of cystic nodal metastasis in p16-positive OPSCC than in p16-negative disease (39.4% versus 18.5%, respectively; p = 0.025), further affirming this association. We also found that necrotic nodal metastasis was significantly more prevalent in p16-negative patients with OPSCC than in p16-positive patients (75.8% versus 51.5%, p = 0.027). The pathophysiology of how OPSCC regional metastatic nodes harboring cystic or necrotic foci develop remains to be determined. In a review of metastatic nodes in neck dissection specimens of patients with squamous cell carcinoma of Waldeyer’s ring origin, Regauer et al. [18] found two histologic forms of nodal cavitation. The first appeared as irregular complex cavities filled with debris and lined with polypoid and papillary malignant epithelium with endophytic growth, high mitotic rates, and incomplete keratinization. The second appeared as cavities with smooth and regular outlines that contained homogenous eosinophilic fluid. It has been suggested that the first form is secondary to pseudocystic changes resulting from spontaneous degradation of keratin and cellular debris within carcinomatous lymph node deposits or from the blockage of lymphatic fluid flow passing through a metastatic node [8,19]. The mechanism of the second form may be secondary to true cystic metastases originating from malignant salivary type cells or transformed keratinocytes, which have intrinsic properties for cyst formation [18,20–22]. However, our patients were treated with chemoradiotherapy instead of surgery; hence, no histopathological correlation with neck metastatic nodes could be obtained. We speculate that our necrotic metastatic nodes may be a result of the complex pseudocystic changes corresponding to the first histologic form of Regauer et al.’s study, whereas the cystic metastatic nodes we observed may be true cystic cavities corresponding to the second histologic form. Further investigations are required to confirm our speculation.

Lymph node status is of great prognostic significance in patients with HNSCC. Most OPSCCs exhibit neck metastases at presentation; this portends decreased disease control and survival. On imaging, metastatic lymphadenopathy may appear as solid, necrotic, or cystic [3]. Intranodal necrosis is visualized on CT as a non-enhancing hypodense area, and such necrotic areas are thought to contain less oxygen and have poorer responses to chemotherapy or radiotherapy. Some studies showed that hypodense metastatic nodes on contrast-enhanced CT had a significantly poorer response to chemotherapy or chemoradiotherapy compared to isodense metastatic nodes [11,12,23], while other studies did not [13,24]. Such inconsistent results may be attributed to the fact that not all the hypodense metastatic nodes were necrotic, as some may have been cystic nodes. Although cystic nodes also present as non-enhancing hypodense areas on CT, they are distinct from necrotic nodes in terms of histology and etiology [18–22]. Different proportions of cystic and necrotic nodal metastases may lead to different treatment responses; therefore, they should be analyzed separately. As seen in the present analysis, we found that necrotic nodal metastasis was independently correlated with regional failure, showing approximately a seven-fold increased risk (OR = 7.31). Consequently, those OPSCC patients with necrotic nodal metastases at presentation should be closely followed up their neck statuses after chemoradiotherapy for early detection of potentially salvageable lesions. Alternatively, they may serve as suitable candidates for planned neck dissection or intensification of chemoradiotherapy. In terms of distant failure, our study showed that advanced nodal stage was an independent elevated risk factor (OR = 4.11), whereas cystic nodal metastasis was an independent reduced risk factor (OR = 0.09). Therefore, distant sites should be carefully monitored in patients with OPSCC who have advanced nodal stage, particularly those without cystic nodal metastases. Since the lung was the most common site of distant failure, extended-field head and neck CT to include the chest may be a preferred follow-up imaging technique in this patient subgroup.

This study had some limitations. First, our patients underwent chemoradiotherapy without neck dissection; hence, histologic evaluation of neck lymph nodes was not available. The exact biological mechanisms that link cystic nodal metastasis to HPV infection and chemoradiotherapy response remain undetermined. Second, p16 immunohistochemistry and viral DNA detection (with polymerase chain reaction or in situ hybridization) are widely used techniques to assess HPV status. However, these tests have their own specific limitations. p16 immunohistochemistry is a highly sensitive technique and provides proof of transcriptional activity, however, it lacks high specificity and is not exclusively linked to HPV infection. HPV DNA detection tests have high specificity but lack sensitivity and information about transcriptional activity. Ideally, all samples should be tested for both p16 and HPV DNA, and only those that are positive in both assays should be considered HPV-related. RNAscope is a novel technology for HPV testing with a high sensitivity and specificity; however, it has not yet been clinically validated [25]. In our study, only p16 immunohistochemistry was used for OPSCC patient stratification, and our patients are therefore described as either p16-positive or p16-negative. Such a stratification system ought to be clinically acceptable, as p16 overexpression was chosen as the proxy of HPV-associated cancer in the recently published 8th edition of the American Joint Committee on Cancer staging manual because of its low cost, universal applicability, and strong correlation with patient survival [7]. Third, all the participants came from a geographic area in which the oropharyngeal HPV infection rate is low, whereas betel quid chewing is endemic [26]. Therefore, our results may not be generalizable to patients in different geographic locations. Furthermore, our study is a retrospective analysis of patients who were treated at a single center; retrospective data analyses such as these may harbor selection biases. Consequently, our results ought to be confirmed and validated in future prospective studies.

## Conclusion

Our study showed that cervical cystic metastatic nodes observed on pretreatment MRI were prevalent in p16-positive patients with OPSCC, while cervical necrotic metastatic nodes were prevalent in p16-negative patients. The presence of cervical necrotic nodal metastasis at presentation was significantly correlated with an increased risk of regional failure. The risk of distant failure increased with advanced nodal stage but decreased with cervical cystic nodal metastasis. On posttreatment follow-up, close attention ought to be paid to the neck region for patients with OPSCC who exhibit necrotic nodal metastases, as well as to distant sites in patients with advanced nodal stages but without cystic nodal metastasis.
